# Efficacy of Pre-operative Physical Therapy on Total Knee Replacements: A Systematic Review of Randomized Controlled Trials

**DOI:** 10.1007/s43465-026-01744-y

**Published:** 2026-02-26

**Authors:** Angel Arturo Rojas, Yumeng Li

**Affiliations:** Department of Health and Human Performance, Texas State University, San Marcos, USA

**Keywords:** Total knee arthroplasty, Prehabilitation, Physiotherapy, Osteoarthritis, Post-operative outcomes

## Abstract

**Purpose:**

The purpose of the study was to determine whether pre-operative physical therapy improves post-operative outcomes in adults undergoing primary unilateral total knee arthroplasty (TKA) for osteoarthritis.

**Methods:**

We conducted a systematic search of MEDLINE, CINAHL, EMbase, Cochrane CENTRAL, and Google Scholar. Eligible studies were randomized controlled trials including adults awaiting primary unilateral TKA that compared a structured pre-operative physical therapy program against usual care/no intervention, initiated ≥ 3 weeks pre-operatively, and reported ≥ 2 post-operative functional outcomes. Two reviewers extracted study characteristics, interventions, and outcomes. Methodological quality was appraised using the PEDro scale. Because of heterogeneity of interventions and outcome measures, a narrative synthesis was performed.

**Results:**

Eleven randomized controlled trials met inclusion criteria with generally good methodological quality. Interventions commonly included lower-limb strengthening, with variable incorporation of balance/proprioception and aerobic warm-up. Across trials, pre-operative physical therapy demonstrated small-to-moderate advantages for early post-operative pain and function (e.g., WOMAC subscales) and for selected performance tests (e.g., timed up and go), while effects on range of motion and length of stay were minimal or inconsistent. Higher-intensity resistance training and programs including proprioceptive work tended to yield more favorable early outcomes. Robust between-group differences at ≥ 6–12 months were uncommon.

**Discussion:**

Pre-operative physical therapy may confer modest, clinically relevant improvements in early pain, function, and selected performance outcomes after TKA, particularly when programs emphasize progressive strengthening and proprioceptive/balance training. Evidence remains heterogeneous, and durable long-term benefits are uncertain. Standardized, adequately powered trials using common outcome sets and evidence-based dosing are needed.

## Introduction

The prevalence of obesity in the USA has risen from 13.4% in 1960–1962 to 38% in 2013–2014 and 42.4% in 2017–2018 [1, 2]. Because obesity is a major risk factor for knee osteoarthritis (OA) and disability, this trend has coincided with sustained growth in joint replacement procedures, with total knee arthroplasty (TKA) now the most common arthroplasty performed [3]. Beyond mechanical load, obesity is associated with systemic and psychosocial factors that can compound functional limitations, highlighting the need for strategies that address both physiological and behavioral contributors to outcomes [3].

Knee OA is a painful, degenerative joint disease characterized by progressive loss of articular cartilage and related changes in subchondral bone and periarticular tissues [4]. Its clinical impact is considerable: OA reduces participation in regular physical activity, interferes with personal care, and diminishes health-related quality of life. Prevalence estimates suggest that approximately 14 million Americans have knee OA [5]. Disease burden and functional loss vary across individuals, influenced by symptom severity, joint damage, comorbidities, and physical conditioning.

Non-operative care remains first-line and includes weight reduction through diet, structured exercise, and patient education [3, 6], complemented by adjunctive modalities such as heat or cold therapy, soft tissue techniques, neuromuscular electrical stimulation, and pharmacologic management [7–10]. Although pain frequently discourages activity, appropriately dosed exercise programs are safe and effective for reducing pain and improving strength and function in knee OA [3, 11, 12]. A review by Raposo and colleagues reported that Pilates, aerobic and strengthening programs, and aquatic interventions delivered over 8–12 weeks reduced patient-reported pain [13]. For individuals with end-stage disease or inadequate response to conservative care, TKA is indicated and generally successful for relieving pain and improving function and quality of life [3]. Nevertheless, persistent post-operative performance deficits compared with individuals without knee pathology are not uncommon [7]. The USA currently has the highest incidence of TKA worldwide, and procedure volumes are projected to grow by approximately 85% by 2030 [14].

Given the substantial burden of knee OA on individuals and health systems, recent efforts have focused on comprehensive, multimodal strategies to optimize care across the peri-operative pathway [7–10]. Pre-operative functional status is a consistent predictor of post-operative recovery: greater quadriceps strength and knee extension before surgery are associated with better early performance on the timed up and go and stair climb test [11, 15–17], and lower pre-operative scores on patient-reported measures such as WOMAC or KOOS predict poorer outcomes after TKA [3, 6, 10–12]. These observations underpin growing interest in pre-operative physical therapy as a means to enhance functional capacity and prepare patients for the physiological stress and transient immobility following surgery [7, 18].

Pre-operative physical therapy typically comprises a brief warm-up, flexibility work, progressive muscle strengthening, neuromuscular and balance training, and recovery modalities, often coupled with education to set expectations and support adherence [6]. Despite its biological plausibility, the effectiveness of pre-operative physical therapy before TKA remains uncertain. Trials vary widely in content, intensity, supervision, duration, and reporting of adherence. Several prior reviews have mixed hip and knee arthroplasty populations or included heterogeneous designs, complicating interpretation. Moreover, many randomized controlled trials do not clearly anchor resistance training to established principles such as the American College of Sports Medicine’s guidelines, limiting reproducibility and dosing transparency [7, 12, 18].

In view of these uncertainties, a focused synthesis of randomized controlled trials is warranted. The purpose of this systematic review is to evaluate whether pre-operative physical therapy improves post-operative outcomes in adults undergoing primary unilateral TKA. Specifically, we examine the effects on patient-reported pain and function, objective performance tests, range of motion, strength, complications, and length of stay, and we explore whether program features, such as progressive resistance and balance training, are associated with more favorable results.

## Methods

### Design

This review was planned a priori and reported in accordance with PRISMA 2020 guidance.

### Eligibility Criteria

We included studies that met all of the following criteria: (1) randomized controlled trial (parallel-group design) with an experimental pre-operative physical therapy group and a usual-care/no-intervention control; (2) adults awaiting primary unilateral total knee arthroplasty (TKA) for osteoarthritis; (3) a structured pre-operative physical therapy program initiated ≥ 3 weeks pre-operatively; and (4) reporting ≥ 2 post-operative functional outcomes (patient-reported and/or performance-based). We excluded non-randomized designs, non-English publications, conference abstracts without full text, studies where hip and knee data could not be separated unless a clearly defined TKA subgroup with extractable data was reported, revision TKA, bilateral or unicompartmental arthroplasty without extractable unilateral data, and duplicate publications.

### Information Sources and Search Strategy

A comprehensive electronic search was conducted from December 2023 to March 2024 using databases: MEDLINE, CINAHL, EMbase, Cochrane CENTRAL, and Google Scholar. Searches were limited to 2010 onward to reflect contemporary surgical and rehabilitation practice and to English-language publications. We combined controlled vocabulary and keywords related to the population, intervention, and procedure, including: *prehabilitation*, *pre-operative*, *preoperative*, *physical therapy*/*physiotherapy*, *arthroplasty*, *replacement*, *knee*, *TKA*, and *TKR*. Boolean operators (AND/OR), truncation, and proximity operators were used as appropriate. Reference lists of included studies were hand searched, and forward citation tracking was performed to identify additional trials.

### Selection Process

**Fig. 1:**
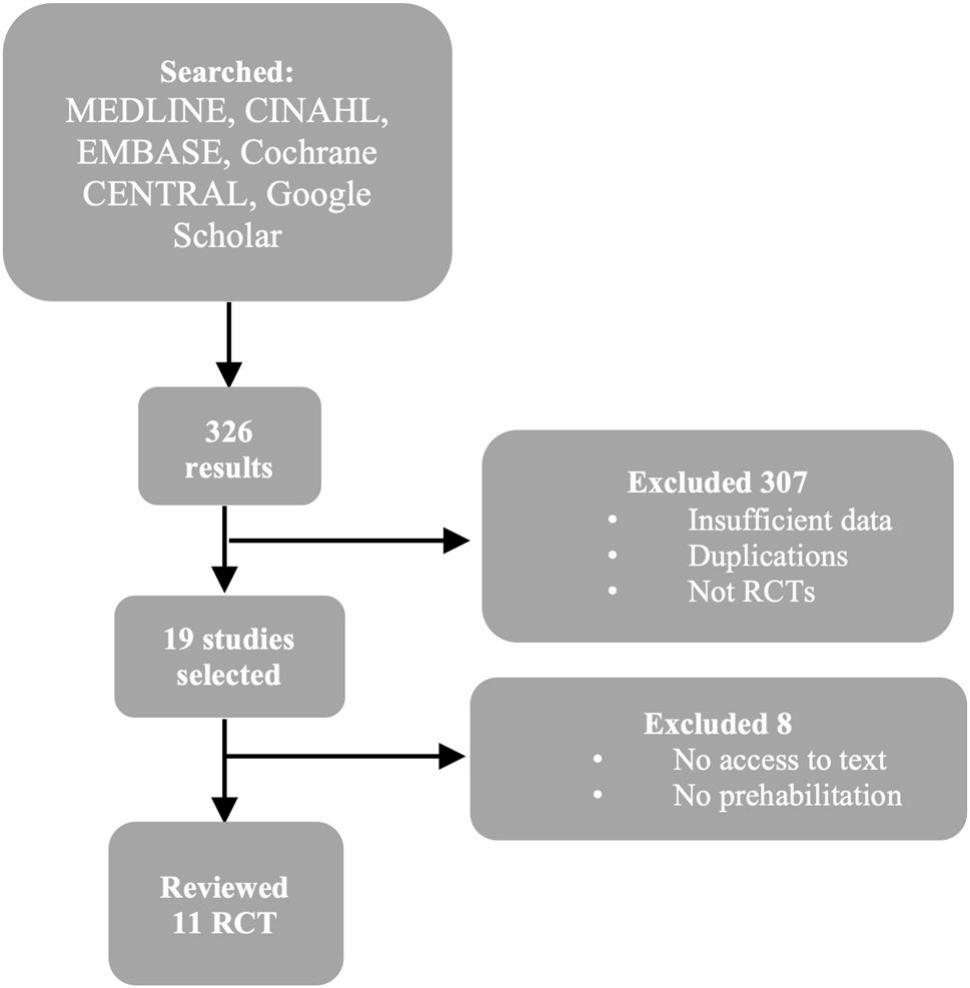
Flowchart of the selection process

Two reviewers independently screened titles/abstracts and then full texts against the eligibility criteria. Discrepancies were resolved by discussion; a third reviewer adjudicated if consensus could not be reached. Reasons for full-text exclusion were recorded. The study selection process is depicted in Fig. 1.

### Data Extraction

Using a piloted spreadsheet, two reviewers independently extracted study characteristics and outcomes, with cross-checking for accuracy. Extracted items included: authors, year, country, sample size, mean age and sex distribution, inclusion/exclusion criteria, surgical laterality, and osteoarthritis diagnosis. For interventions, we captured program frequency, intensity, type, and time; components (warm-up; aerobic/cycling; resistance type and prescription; balance/proprioception; flexibility; neuromuscular electrical stimulation); supervision (supervised vs home-based); setting; adherence/attendance; and comparator description. Outcome domains and time points were extracted as reported. When information was unclear, we consulted supplementary materials or contacted authors when feasible.

### Outcomes

The primary outcomes were patient-reported pain and function (e.g., WOMAC, KOOS). Secondary outcomes included performance tests (e.g., timed up and go, stair climb test), knee range of motion, isometric/isokinetic strength, post-operative complications/adverse events, and length of stay. For synthesis, post-operative assessments were grouped as early (≤ 6 weeks), intermediate (~ 3 months), and late (6–12 months) when multiple time points were available.

### Risk of Bias and Methodological Quality

Methodological quality was assessed with the PEDro scale (11 items; total score 0–10). The 11 items assess: (1) specification of eligibility criteria; (2) random allocation; (3) concealed allocation; (4) baseline comparability; (5) blinding of participants; (6) blinding of therapists; (7) blinding of assessors; (8) adequate follow-up (≥ 85% of participants providing outcome data); (9) intention-to-treat analysis; (10) between-group statistical comparisons; and (11) reporting of point estimates and measures of variability. In line with PEDro conventions, Item 1 is not included in the final score.

Given the nature of exercise trials, blinding of therapists and participants (items 5 and 6) is generally infeasible. These items were systematically rated as “no” and were not overweighted in interpretation. Trials scoring ≥ 6/10 were considered to have acceptable methodological quality, whereas scores < 6/10 were interpreted as indicating higher risk of bias.

**Fig. 2:**
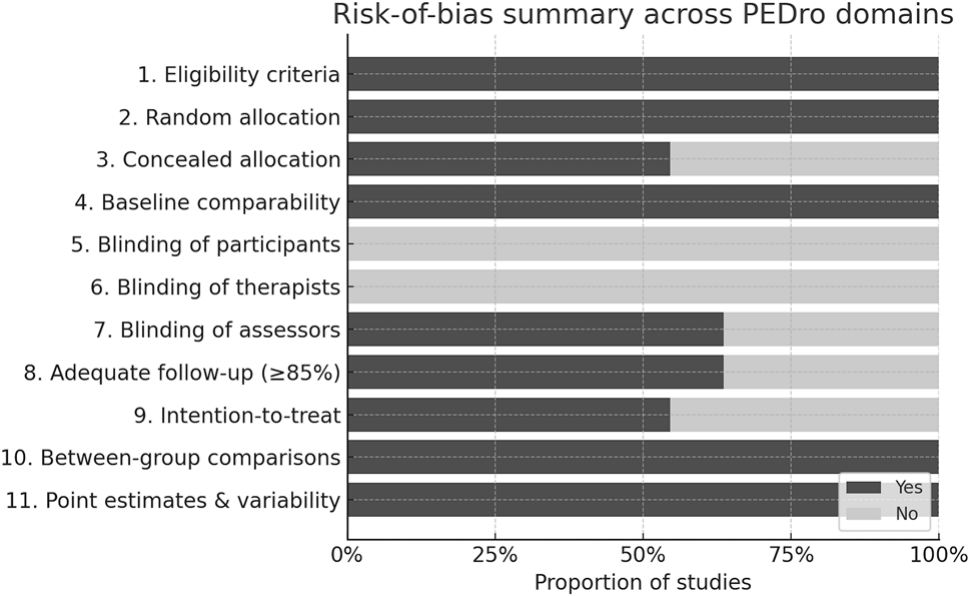
Risk-of-bias summary across PEDro domains

Individual PEDro item ratings and total scores for each trial are summarized in Table 1, along with brief justifications for low-scoring items (e.g., lack of concealed allocation, incomplete follow-up, or absence of intention-to-treat analysis). The trial by Mat Eil-Ismail et al. (2016), which scored 4/10, was downgraded primarily due to unclear or absent allocation concealment, lack of blinded outcome assessment, and incomplete reporting of follow-up and intention-to-treat procedures. A risk-of-bias summary is presented in Fig. 2

**Table 1:** Study-by-study risk of bias assessment using the Physiotherapy Evidence Database scale (PEDro scale)

Study	1	2	3	4	5	6	7	8	9	10	11	Score	Key limitations
Blasco et al. 2020 [11]	Y	Y		Y			Y	Y		Y	Y	6	Unclear ITT
Calatayud et al. 2017 [21]	Y	Y	Y	Y				Y	Y	Y	Y	7	Incomplete ITT
Cavill et al. 2016 [15]	Y	Y		Y			Y	Y	Y	Y	Y	7	
Domínguez-Navarro et al. 2021 [3]	Y	Y	Y	Y			Y			Y	Y	6	Incomplete follow-up/ITT
Gränicher et al. 2020 [16]	Y	Y	Y	Y				Y	Y	Y	Y	7	Incomplete ITT
Mat Eil-Ismail et al. 2016 [8]	Y	Y		Y						Y	Y	4	Unclear allocation concealment, no blinded assessor, incomplete follow-up/ITT
Matassi et al. 2014 [9]	Y	Y		Y			Y	Y		Y	Y	6	
McKay et al. 2012 [10]	Y	Y	Y	Y					Y	Y	Y	6	Limited follow-up/ITT
Nguyen et al. 2022 [12]	Y	Y	Y	Y			Y		Y	Y	Y	7	Incomplete ITT
Skoffer et al. 2016 [17]	Y	Y	Y	Y			Y	Y	Y	Y	Y	8	
Tungtrongjit et al. 2012 [22]	Y	Y		Y			Y	Y		Y	Y	6	Unclear ITT

*ITT* intention-to-treat analysis. Because blinding of participants and therapists was not feasible for exercise-based pre-operative programs, Items 5 and 6 were rated “N” for all studies and were not overweighted when interpreting overall risk of bias. The trial by Mat Eil-Ismail et al. (2016), which scored 4/10, was downgraded primarily because of unclear or absent allocation concealment, lack of blinded outcome assessment, and incomplete reporting of follow-up and intention-to-treat procedures

### Synthesis Methods

We planned a meta-analysis contingent upon sufficient clinical and statistical homogeneity. However, because of heterogeneity in intervention content/dose, comparators, and outcome measures/timing, we conducted a structured narrative synthesis.

Where trials reported means and standard deviations on common scales, we summarized the direction and magnitude of between-group effects and noted whether differences were likely to be clinically important. We also calculated standardized mean differences (Cohen’s *d*). To aid interpretation, we considered standardized mean differences of approximately 0.2, 0.5, and 0.8 as small, moderate, and large, respectively. For WOMAC and KOOS, we interpreted between-group differences relative to published minimal clinically important differences, typically in the range of ~ 10–12 points for WOMAC [19] and ~ 10–20 points for KOOS [20] total or subscale scores, recognizing variation across studies and populations.

Because there were fewer than ten studies contributing to any single outcome domain with comparable measures and time points, we did not formally perform subgroup/meta-regression analyses. No imputation of missing outcome data was undertaken.

## Results

### Study Selection and Characteristics

Eleven randomized controlled trials met the inclusion criteria (Table 2). Most trials included older adults (mean age ≥ 62 years), with a predominance of women in experimental groups. Interventions varied in content and dose, but all programs included lower-limb strengthening; several incorporated flexibility, aerobic warm-ups, or balance/proprioceptive training (Table 3). Methodological quality was generally good (PEDro 6–8/10 in most studies), with one study scoring 4/10 (Table 1).

**Table 2:** Participant characteristics of included randomized controlled trial studies

Study	Group	Sample size	Age (SD)	Sex (% female)
Blasco et al. 2020 [11]	Intervention (hospital based) Intervention (home based) Control	25 26 26	70.2 (7.2) 72.3 (4.5) 70.9 (9.5)	76% 73% 58%
Calatayud et al. 2017 [21]	Intervention Control	22 22	66.8 (4.8) 66.7 (3.1)	84% 84%
Cavill et al. 2016 [15]	Intervention Control	21 20	66.0 (8.4) 68.3 (9.1)	52% 55%
Domínguez-Navarro et al. 2021 [3]	Intervention (strength only) Intervention (strength + balance) Control	24 20 21	70.8 (5.4) 70.4 (6.4) 70.2 (5.6)	58% 65% 67%
Gränicher et al. 2020 [16]	Intervention Control	10 10	66.6 (7.5) 68.1 (7.6)	30% 50%
Mat Eil-Ismail et al. 2016 [8]	Intervention Control	24 26	62.4 64.3	92% 81%
Matassi et al. 2014 [9]	Intervention Control	61 61	66 (7.2) 67 (7.7)	48% 43%
McKay et al. 2012 [10]	Intervention Control	10 12	63.5 (4.9) 60.6 (8.1)	50% 67%
Nguyen et al. 2022 [12]	Intervention Control	131 131	68.2 (7.3) 68.9 (7.7)	70% 66%
Skoffer et al. 2016 [17]	Intervention Control	30 29	70.7 (7.3) 70.1 (6.4)	63% 59%
Tungtrongjit et al. 2012 [22]	Intervention Control	30 30	63.0 (7.6) 65.9 (7.2)	87% 80%

**Table 3:** Intervention content and design

Study	Intervention	Duration (weeks)	# of session	Session duration (min)	Frequency (times per week)
Blasco et al. 2020 [11]	Strength, balance training	12	12	N/A	3
Calatayud et al. 2017 [21]	Dynamic warm-up, strength, step training	8	24	30	3
Cavill et al. 2016 [15]	Step training, functional training, quad training, manual therapy	4	8	60	2
Domínguez-Navarro et al. 2021 [3]	Strength, strength + balance, proprioceptive training	4	12	30–40	3
Gränicher et al. 2020 [16]	Endurance training, PNF stretching, education, strength training	3–4	5–9	N/A	N/A
Mat Eil-Ismail et al. 2016 [8]	Thermotherapy, aerobic training, quad training, stretching	6	N/A	N/A	N/A
Matassi et al. 2014 [9]	Strength, flexibility	6	30	N/A	5
McKay et al. 2012 [10]	Aerobic warm-up, bilateral exercise	6	18	30	3
Nguyen et al. 2022 [12]	Education, strength training, stretching, endurance training, proprioceptive training, balance training	8	16	90	2
Skoffer et al. 2016 [17]	Warm-up, progressive resistance training, stretching	4	12	60	3
Tungtrongjit et al. 2012 [22]	Quad strength training	3	63	N/A	3

### Patient-Reported Outcomes (WOMAC, KOOS)

A summary of outcome results is presented in Table 4. Several trials reported pain and function using WOMAC or KOOS scales. Overall, pre-operative physical therapy was associated with small-to-moderate early improvements in pain and function relative to usual care, particularly on WOMAC subscales, whereas longer-term differences were small and often not statistically significant.

**Table 4:** Summary of outcome results from the included randomized controlled trial studies

Study	Primary outcome measures	Assessment time points post-TKA: intervention group mean (SD) vs. control group mean (SD). (95% CI) if available	*p* Value
Blasco et al. 2020 [11]	Berg balance scale (score)	Hospital intervention: 44.3 (9.1), Cohen’s *d* vs control = − 0.31 Home intervention: 44.2 (7.0), Cohen’s *d* vs control = − 0.34 Control: 47.5 (11.6)	0.497
	KOOS-ADL (%)	Hospital intervention: 30.5 (17.9), Cohen’s d vs control = − 0.51 Home intervention: 35.0 (18.3), Cohen’s *d* vs control = − 0.28 Control: 40.4 (20.6)	0.210
Calatayud et al. 2017 [21]	ROM flexion (deg)	1 month: 88.8 (85.4 to 92.3), 82.3 (78.8 to 85.8), Cohen’s *d* = 0.78 3 month: 101.2 (97.8 to 104.7), 96.4 (92.9 to 99.9), Cohen’s *d* = 0.58	< 0.01*
	ROM extension (deg)	1 month: 11.1 (10.1 to 12.2), 16.9 (15.9 to 17.9), Cohen’s *d* = − 2.36 3 month: 8.2 (7.2–9.3), 13.9 (12.8–14.9), Cohen’s *d* = − 2.27	< 0.01*
	Timed up and go (s)	1 month: 7.3 (6.9–7.6), 9.4 (9.0–9.7), Cohen’s *d* = − 2.51 3 month: 7.0 (6.7–7.3), 8.7 (8.3–9.1), Cohen’s *d* = − 2.01	< 0.01*
	Stair test (s)	1 month: 9.1 (8.4–9.7), 12.7 (12.1–13.4), Cohen’s *d* = − 2.31 3 month: 7.9 (7.2–8.5), 12.1 (11.5–12.8), Cohen’s *d* = − 2.70	< 0.01*
Cavill et al. 2016 [15]	Length of stay (days)	7.3 (2.6), 6.7 (1.9), Cohen’s *d* = 0.26	0.96
	Knee flexion (deg)	110.8 (10.6), 98.2 (11.1), Cohen’s *d* = 1.16	< 0.01*
	Knee extension (deg)	12.9 (8.5), 13.3 (6.4), Cohen’s *d* = − 0.05	0.82
	Timed up and go (s)	11.8 (6.8), 10.5 (5.2), Cohen’s *d* = 0.21	0.72
Domínguez-Navarro et al. 2021 [3]	Berg balance scale (score)	Strength only: 48.0 (4.3), Cohen’s *d* vs control = − 0.07 Strength + balance: 49.5 (3.1), Cohen’s *d* vs control = 0.31 Control: 48.3 (4.4)	0.38
	KOOS-ADL (%)	Strength only: 55.5 (17.8), Cohen’s d vs control = − 0.03 Strength + balance: 56.0 (14.4), Cohen’s d vs control = 0.00 Control: 56.0 (14.4)	0.60
Gränicher et al. 2020 [16]	Stair test (s)	12.58 (4.64), 13.59 (5.30), Cohen’s *d* = − 0.20	0.658
	Tegner activity scale	3.80 (0.79), 2.50 (0.85), Cohen’s *d* = 1.58	0.232
	Length of stay (weeks)	1.0 (1.4), 1.5 (1.6), Cohen’s *d* = − 0.33	0.486
Mat Eil-Ismail et al. 2016 [8]	Pain	6 week: 86.94 (81.06, 92.82), 82.95 (77.30, 88.60), Cohen’s *d* = 0.27 3 month: 94.35 (90.49, 98.23), 88.06 (84.34, 91.77), Cohen’s *d* = 0.65	0.303
	ADL	6 week: 81.58 (75.37, 87.78), 74.52 (68.56, 80.48), Cohen’s *d* = 0.46 3 month: 89.50 (85.04, 93.97), 80.56 (76.27, 84.84), Cohen’s *d* = 0.79	0.205
Matassi et al. 2014 [9]	Days to 90 deg knee flexion	5.8 (2.1), 6.9 (1.9), Cohen’s *d* = − 0.55	< 0.01*
	Length of stay (days)	9.1 (2.1), 9.9 (2.3), Cohen’s *d* = − 0.36	0.011*
McKay et al. 2012 [10]	Quad strength (Nm/kg)	6 week: 0.60 (0.39), 0.57 (0.29), Cohen’s *d* = 0.09 12 week: 0.77 (0.56), 0.74 (0.35), Cohen’s *d* = 0.07	> 0.05
	50-foot walk (s)	6 week: 14.23 (7.55), 13.11 (3.30), Cohen’s *d* = 0.20 12 week: 11.80 (5.66), 11.82 (2.96), Cohen’s *d* = 0.01	> 0.05
	WOMAC pain	6 week: 5.60 (2.72), 4.92 (4.50), Cohen’s *d* = 0.18 12 week: 4.40 (3.20), 3.58 (4.40), Cohen’s *d* = 0.21	> 0.05
Nguyen et al. 2022 [12]	Pain intensity (0–100)	6 month: 24.5 (21.4), 25.7 (23.2), Cohen’s *d* = − 0.05 12 month: 25.9 (26.5), 23.1 (21.8), Cohen’s *d* = 0.12	0.86 0.30
	WOMAC function (0–100)	6 month: 47.1 (19.7), 49.7 (18.1), Cohen’s *d* = − 0.14 12 month: 44.9 (20.2), 48.3 (19.4), Cohen’s *d* = − 0.17	0.26 0.33
	No. of steps in the past week	6 month: 3981 (2061), 3712 (2161), Cohen’s *d* = 0.13 12 month: 4969 (3481), 4600 (3757), Cohen’s *d* = 0.10	0.84 0.56
Skoffer et al. 2016 [17]	10 m walking (s)	1 week: 12.5 (4.9), 14.4 (5.6), Cohen’s *d* = − 0.36 6 week: 7.6 (1.8), 8.6 (1.6), Cohen’s *d* = − 0.59 12 week: 7.1 (1.5), 7.7 (1.2), Cohen’s *d* = − 0.44	0.225 0.119 0.216
	30-s chair stand test (rep)	1 week: 4.4 (5.1), 2.2 (3.5), Cohen’s *d* = 0.50 6 week: 13.3 (5.0), 9.6 (4.4), Cohen’s *d* = 0.78 12 week: 14.7 (4.7), 11.0 (4.4), Cohen’s *d* = 0.79	0.116 0.004* 0.001*
	Timed up and go (s)	1 week: 14.8 (5.2), 17.0 (5.6), Cohen’s *d* = − 0.41 6 week: 8.3 (2.3), 10.0 (2.4), Cohen’s *d* = − 0.72 12 week: 7.9 (2.3), 8.9 (2.1), Cohen’s *d* = − 0.45	0.044* 0.015* 0.050*
Tungtrongjit et al. 2012 [22]	Pain score	1 month: 2.9 (1.5), 3.8 (1.4), Cohen’s *d* = − 0.62 3 months: 1.6 (1.3), 2.6 (1.4), Cohen’s *d* = − 0.74 6 months: 0.9 (1.4), 1.4 (1.3), Cohen’s *d* = − 0.37	0.032* 0.003* 0.137
	WOMAC	1 month: 62.6 (25.3), 89.3 (30.1), Cohen’s *d* = − 0.96 3 months: 31.2 (22.2), 48.9 (26.1), Cohen’s *d* = −0.73 6 months: 18.7 (17.9), 22.0 (14.1), Cohen’s *d* = −0.20	< 0.01* < 0.01* 0.160
	Quad strength (kg)	1 month: 5.5 (2.9), 4.0 (2.7), Cohen’s *d* = 0.54 3 months: 7.5 (2.9), 5.3 (3.4), Cohen’s *d* = 0.70 6 months: 8.4 (3.4), 7.2 (4.0), Cohen’s *d* = 0.32	0.012* < 0.01* 0.067

* indicates statistical significance (*p* < 0.05)

For WOMAC pain and function, several trials showed between-group differences favoring pre-operative physical therapy at early follow-up (≤ 6 weeks to ~ 3 months), with effect sizes typically in the small-to-moderate range (Cohen’s *d* = 0.03–0.79). In a representative trial by Tungtrongjit et al., WOMAC pain scores improved in both groups, but with a faster trajectory and larger absolute change in the intervention group at 1–3 months post-TKA. Across studies, most between-group differences did not consistently exceed commonly published minimal clinically important differences for WOMAC or KOOS (~ 10–12 points for WOMAC and ~ 10–20 points for KOOS), although some early time points approached these thresholds.

Trials using KOOS tended to report improvements in both groups with minimal or no between-group differences at 6–12 months, consistent with the notion that pre-operative care and standard rehabilitation attenuate early advantages over time. The consistency of findings across WOMAC and KOOS suggests that pre-operative physical therapy confers modest early benefits in symptoms and self-reported function, but does not clearly alter longer-term trajectories.

The strength of evidence for patient-reported outcomes is moderate: most contributing trials had acceptable PEDro scores (≥ 6/10), sample sizes were modest, and blinding of participants/therapists was not feasible. The single low-quality trial (PEDro 4/10) contributed limited estimates and did not change the direction of the overall pattern. Thus, while risk of bias (particularly in allocation concealment and blinding of assessors) may have influenced some effect estimates, the early and modest benefits appear reasonably robust.

### Performance-Based Outcomes

Several trials assessed objective performance, most commonly the timed up and go and stair climb tests. Pre-operative physical therapy generally favored better early performance, particularly for programs that emphasized progressive resistance and/or balance training. Effect sizes were small to moderate and most evident in the first 1–3 months post-operatively. For example, Skoffer et al. reported a small to moderate differences (Cohen’s *d* = 0.36–0.78) in favor of progressive resistance training for performance-based outcomes at 1–6 weeks, which persisted as a modest advantage at 12 weeks (Cohen’s *d* = 0.44–0.79).

Not all trials found significant or clinically important between-group differences, especially when interventions were lower-intensity or adherence was suboptimal. Stair climb findings were mixed, with some studies showing small early gains that were not sustained beyond 3 months.

The performance-based evidence is based on a smaller number of trials with generally acceptable PEDro scores. However, incomplete blinding of assessors and occasional loss to follow-up introduce some risk of bias. Overall, the evidence suggests that pre-operative physical therapy, particularly when delivered at higher intensity with progressive resistance, may yield modest improvements in early functional performance, but the durability and clinical significance of these gains remain uncertain.

### Knee Range of Motion

Most trials reported post-operative knee flexion and/or extension. Pre-operative physical therapy had minimal influence on final ROM. Most between-group differences in flexion or extension were small and not statistically significant. Where advantages were present, they were typically in the range of only a few degrees.

In Blasco et al., a multimodal program (proprioception, gait training, isometric/resistance) produced small, non-significant flexion advantages at 2 and 6 weeks. Domínguez-Navarro et al. observed numerically greater flexion in the strength + balance group versus control at 6 weeks, with a smaller effect for strength-only training, but neither difference reached statistical significance. Some trials noted that pre-operative physical therapy participants achieved functional flexion earlier than controls despite similar absolute ROM later, suggesting a possible shift in early recovery rather than in final joint mobility.

Overall, the consistency of findings across moderate-quality trials indicates that pre-operative physical therapy does not substantially alter long-term ROM outcomes. The strength of evidence is moderate, and the risk of bias related to ROM measurement is low, because goniometric assessment is relatively objective and assessors were sometimes blinded.

### Muscle Strength

Several trials reported isometric or isokinetic strength outcomes. Pre-operative physical therapy generally improved quadriceps and/or hamstring strength, with high-intensity protocols showing the largest gains. In Tungtrongjit et al., high-intensity intervention produced substantial strength improvements and maintained a moderate between-group advantage at 1–6 months post-operation (Cohen’s *d* = 0.32–0.70). Skoffer et al. similarly reported a small but statistically significant between-group difference in isometric knee flexion at 12 weeks favoring progressive resistance training.

Trials using moderate-intensity or predominantly flexibility-based programs reported smaller, often non-significant between-group differences, suggesting a dose–response relationship. Programs that did not explicitly progress load or did not meet recognized resistance-training principles tended to produce limited strength transfer.

Strength outcomes were derived from a smaller subset of trials, but most had acceptable methodological quality. Measurement procedures were typically standardized, reducing measurement bias. However, incomplete reporting of adherence and progression limits certainty regarding the optimal dose and format of intervention to maximize strength benefits.

### Length of Stay and Complications

Hospital length of stay was reported in several trials. Between-group differences in length of stay were consistently small (often < 1 day) and rarely statistically significant. For example, Gränicher et al. observed a non-significant reduction of approximately half a day in length of stay in the intervention group, while Cavill et al. reported virtually identical length of stay between groups despite added neuromuscular and stretching components.

Post-operative complications and adverse events were infrequently reported. No study provided evidence of increased harm associated with pre-operative physical therapy.

The length of stay and complication data provide low-to-moderate strength evidence that pre-operative physical therapy does not materially change hospital stay or complication rates, although reporting was often incomplete and not always pre-specified.

### Summary of Effects by Time Window

Taken together, the evidence suggests modest, clinically relevant early benefits (≤ 6 weeks to ~ 3 months) of pre-operative physical therapy on pain, function, and selected performance tests, with minimal effects on ROM, length of stay, and uncertain durability at 6–12 months. Programs emphasizing progressive resistance and proprioceptive/balance training tended to show more consistent early advantages [3, 11, 17, 21].

The overall strength of evidence is moderate for early patient-reported and performance outcomes and low to moderate for ROM, length of stay, strength, and complications. Risk of bias (particularly allocation concealment and incomplete follow-up) may have inflated some early effect estimates, but is unlikely to fully account for the pattern of small, short-term benefits.

## Discussion

Across 11 randomized controlled trials, pre-operative physical therapy produced modest, clinically meaningful early benefits in patient-reported pain and function (≤ 6 weeks to ~ 3 months), with occasional advantages on performance tests such as the timed up and go and stair climb test. In contrast, effects on knee ROM and length of stay were small and generally not significant, and between-group differences often attenuated by 6–12 months [3, 10–12, 17, 22]. Programs that deployed progressive resistance at moderate-to-high intensity and/or incorporated proprioceptive/balance training tended to demonstrate the most consistent early advantages [3, 17, 21].

The pattern of findings is compatible with the mechanistic rationale for pre-operative physical therapy. Knee OA is characterized by quadriceps weakness and impaired sensorimotor control. Strengthening and proprioceptive training can improve pre-operative capacity and may buffer the immediate post-operative decline. Reviews of exercise for knee OA similarly report reductions in pain with strengthening, aerobic, Pilates, and aquatic interventions over 8–12 weeks [13]. However, heterogeneity in content and dosing likely diluted effect estimates across trials. Several studies applied low-to-moderate intensity or predominantly flexibility-based programs and reported limited strength transfer, whereas high-intensity, progressive resistance yielded clearer strength benefits pre- and post-TKA [10, 12, 21]. Many trials provided incomplete reporting of prescription or did not explicitly align prescription to established principles (e.g., ACSM), limiting reproducibility and dose–response inference [7, 12, 18].

Pre-operative physical therapy appears safe and can enhance early post-operative recovery when it includes: (1) a structured warm-up; (2) progressive lower-limb resistance training (2–3 sessions/week with planned progression); and (3) balance/proprioceptive training; ideally coupled with education to set expectations and support adherence [3, 11, 17]. Patients with lower baseline function may benefit the most. Clinicians should counsel patients that benefits are expected to be largest in the first 1–3 months, may diminish thereafter, and that length of stay is unlikely to change significantly [9, 15, 16].

Pre-operative physical therapy before TKA offers modest early improvements in pain, function, and selected performance measures, particularly when programs emphasize progressive resistance and proprioceptive/balance training. Long-term superiority over usual care remains unproven, and effects on ROM and length of stay are minimal. While current evidence does not mandate universal pre-operative physical therapy, a targeted, adequately dosed program is reasonable for patients with low baseline function and for centers aiming to enhance early recovery while maintaining realistic expectations [3, 10, 11, 21]. It should be noted that pre-operative physical therapy complement, not replace, standard enhanced recovery after surgery and post-operative rehabilitation pathways.

Most included trials had acceptable methodological quality by PEDro (6–8/10), with one outlier scoring 4/10. However, important limitations remain. Samples were modest, adherence variably reported, and blinding of participants/therapists was infeasible. This review was also limited by substantial clinical and methodological heterogeneity across trials. Interventions differed in duration, intensity, supervision, and inclusion of balance or neuromuscular components. Comparators ranged from usual care to enhanced education. Outcome instruments and follow-up time points were not standardized. These differences, along with incomplete reporting of dispersion statistics in some trials, precluded meta-analysis despite the calculation of individual effect sizes. In addition, one included pilot trial randomized both total hip and knee arthroplasty candidates. We restricted our extraction to the TKA subgroup, using joint-specific data where reported. However, the mixed joint sampling and relatively small TKA subgroup introduce potential selection bias. Lastly, follow-up rarely exceeded 12 months, limiting conclusions on durability.

Future trials should: (1) standardize outcome sets (e.g., WOMAC/KOOS, timed up and go, and stair climb test) at common, pre-specified time points (e.g., early, 3 months, 6–12 months) and report minimal clinically important differences; (2) use detailed TIDieR-level intervention reporting with explicit prescription and progression rules, aligned to recognized resistance-training principles; (3) compare intensity-matched protocols (e.g., high vs moderate intensity) and the incremental value of proprioceptive/balance training; (4) pre-register protocols, apply contemporary risk-of-bias methods, and ensure adequate power; (5) include patients with low pre-operative function and explore supervised vs home-based delivery; and (6) assess long-term outcomes (> 12 months) and cost-effectiveness.

## Conclusions

Pre-operative physical therapy before TKA confers modest, clinically meaningful early benefits in pain, function, and selected performance outcomes, particularly when programs emphasize progressive resistance and proprioceptive/balance training. Effects on knee ROM and hospital length of stay are small, and consistent superiority beyond 6–12 months remains uncertain. Given its safety and biological plausibility, pre-operative physical therapy can be considered as an adjunct to standard care, especially for patients with low pre-operative function, provided that programs are adequately dosed, progressed, and supported by education. Future trials should adopt common, pre-specified outcome sets, report prescription and adherence in detail, compare intensity-matched protocols, and extend follow-up to clarify long-term effectiveness and cost-effectiveness.

## Data Availability

This article is a systematic review of previously published studies. All data extracted and analyzed in this review were obtained from the articles cited in the reference list.
